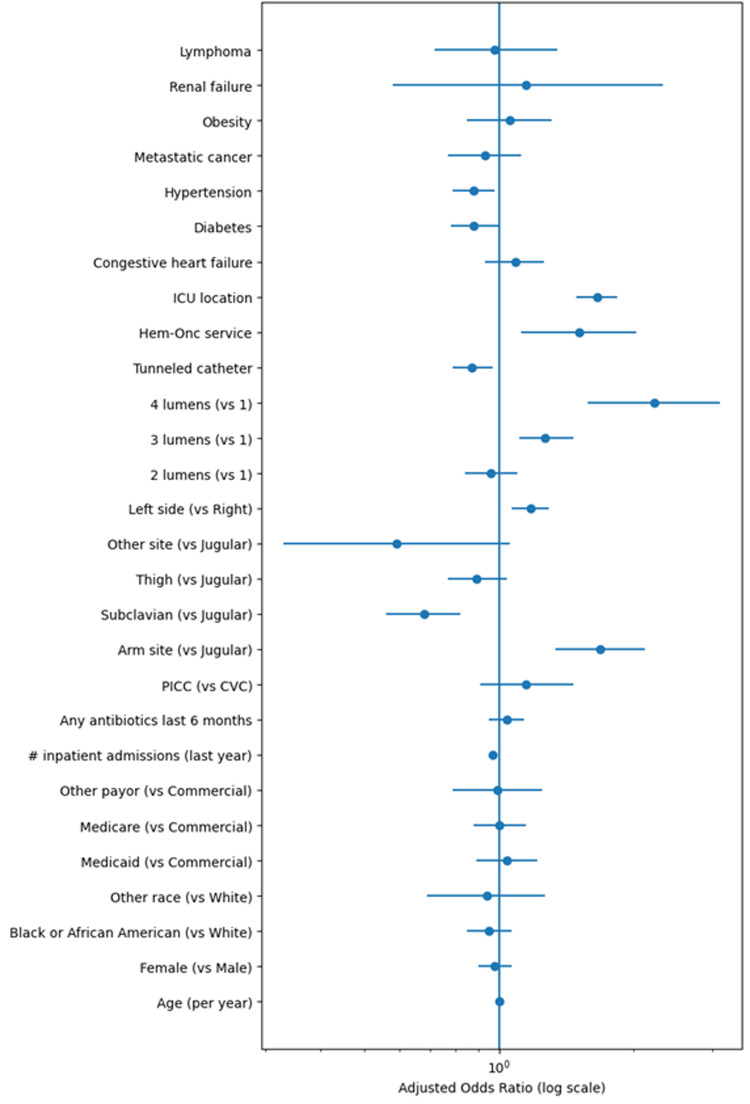# 168 Characteristics of NDM-CRE clusters in the US: Using cluster size dispersion to estimate the probability of final cluster size

**DOI:** 10.1017/ash.2026.10569

**Published:** 2026-06-23

**Authors:** Alexia El Khoury, Joy Abou Farah, Luke Kabbara, Ethan Martin, Zainab Albar, Tina Lewis, Jay Krishnan, Elie Saade, University Hospitals Cleveland Medical Center

**Affiliations:** 1 Case Western Reserve University; 2 Case Western Reserve University/ UH Hospitals; 3 University Hospitals, Cleveland; 4 University Hospitals Health System; 5 University Hospitals, Case Western Reserve University

## Abstract

**Background:** Nasal colonization with Staphylococcus aureus is a well-established risk factor for central line–associated bloodstream infections (CLABSIs). Many institutions have adopted screening and targeted decolonization strategies using intranasal mupirocin; however, real-world implementation remain incompletely characterized. **Methods:** We conducted a retrospective cohort study at University Hospitals Cleveland Medical Center to evaluate implementation of S. aureus screening and decolonization protocol among hospitalized adults undergoing central venous catheter (CVC) or peripherally inserted central catheter (PICC) placement. All patients aged ≥18 years admitted between October 1, 2023, and May 5, 2025, who had a catheter placed were included. Patients with documented bloodstream infection at the time of catheter placement were excluded. Primary implementation outcomes were: (1) proportion of patients screened for S. aureus colonization, and (2) proportion of colonized patients who received prophylactic intranasal mupirocin. Secondary outcomes included overall mupirocin use, bloodstream infection (BSI), and CLABSI rates. Mixed effects logistic regression was performed to identify factors associated with screening uptake. **Results:** Among 11,107 central lines placed, 63.4% underwent S. aureus screening. Of those screened, 22.6% tested positive for Staph aureus. Only 21.2% of colonized patients received intranasal mupirocin, corresponding to an overall mupirocin use rate of 5.3%. Across all central lines placed during the study period, the overall BSI and CLABSI rates were 0.91% and 0.67%, respectively. In adjusted analyses, screening was more likely among patients with arm insertion sites (OR 1.68, 95% CI 1.34–2.12), left-sided catheter (OR 1.18, 95% CI 1.07–1.29), increasing catheter lumens (three lumens: OR 1.27, 95% CI 1.11–1.47; four lumens: OR 2.22, 95% CI 1.58–3.12), ICU location (OR 1.66, 95% CI 1.49–1.84), and hematology–oncology services (OR 1.51, 95% CI 1.12–2.03). Although catheter type itself was not independently associated with screening, patients receiving PICCs demonstrated a positive, though non-significant, trend toward higher screening uptake compared with CVCs (OR 1.15, 95% CI 0.91–1.47). Tunneled catheters (OR 0.87, 95% CI 0.79–0.97) and a higher number of prior inpatient admissions were associated with lower odds of screening, while demographic characteristics and most comorbidities were not. **Conclusions:** S. aureus screening uptake among patients with central lines was low, and gaps at subsequent steps in the process resulted in fewer than one quarter of colonized patients receiving mupirocin. This stepwise breakdown in implementation highlights opportunities to improve protocol reliability by reducing decision points through more standardized approaches.

**Figure:**
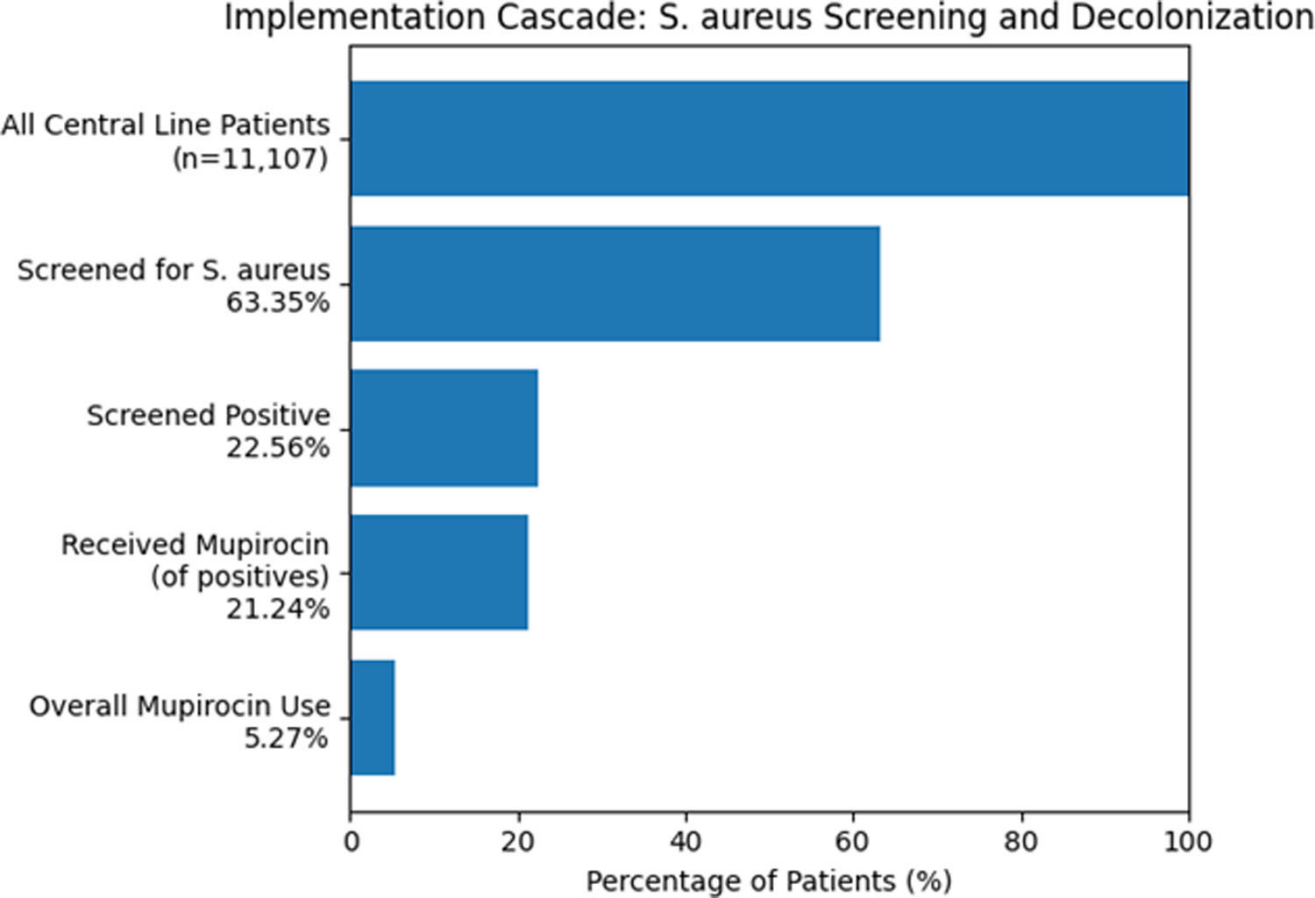

**Figure:**